# Transcranial Endovascular Embolization of Dural Arteriovenous Fistula: A Case Report and Literature Review

**DOI:** 10.7759/cureus.65032

**Published:** 2024-07-21

**Authors:** Ali Abu-Alya, Azaam Mamoor, Devraj Chavda, Erek Helseth, Venkatachalam Veerappan, John A Anson, Mohamad Fayad

**Affiliations:** 1 Neurology, Hospital Corporation of America (HCA) Southern Hills Hospital, Las Vegas, USA; 2 Epilepsy, Columbia University, New York, USA; 3 Pediatric Neurology, State University of New York Downstate Medical Center, Brooklyn, USA; 4 Interventional Neuroradiology, Hospital Corporation of America (HCA) Southern Hills Hospital, Las Vegas, USA; 5 Neurosurgery Department, Hospital Corporation of America (HCA) Southern Hills Hospital, Las Vegas, USA

**Keywords:** spontaneous intracerebral hemorrhage, intracerebral hematoma, craniotomy, endovascular embolization, dural arteriovenous fistulas

## Abstract

The optimal endovascular management of high-grade dural arterial-venous fistulae (dAVF) can be technically challenging in cases where the involved venous sinus segment is inaccessible through the traditional percutaneous approach. In this report, we describe our experience with the transcranial endovascular approach for the treatment of a high-grade dAVF. We also provide a literature review of other reports of transcranial endovascular dAVF embolization. We propose that transcranial endovascular embolization of high-grade dAVF appears to be safe and effective in cases where the fistula is inaccessible to percutaneous routes alone.

## Introduction

Intracranial dAVF are abnormal connections between arteries and dural sinuses or veins within the dural leaflets [1]. They are commonly graded based on the Borden and Cognard classification schemes [2,3]. Low-grade fistulae can be incidental but should be monitored clinically as they occasionally undergo grade-up conversion [4]. Fistulae are characterized as high-grade when they develop cortical venous drainage (CVD) due to venous hypertension and sinus outflow obstruction [1-3]. High-grade fistulae can cause intracranial hemorrhage or significant non-hemorrhagic neurologic deficits (NHND) due to venous hypertension with resultant cerebral edema [1]. High-grade fistulae have an annual rupture rate of 15-35%; thus, they require treatment [4]. 

The first line of treatment for symptomatic high-grade dAVF is endovascular embolization [4-6]. This can be done through trans-arterial embolization (TAE), trans-venous embolization (TVE), or a combination of both. The goal of therapy is to completely obliterate the fistula to correct venous shunting [5,6]. In TAE, a microcatheter is navigated into the feeding arteries, and embolic material is injected with the goal of occluding the distal feeding arteries with penetration of the fistulous point in the draining vein or sinus [5,6]. This is challenging to accomplish in cases that have multiple fistulous points, feeding arteries that have extensive complex collateralization, arteries that supply eloquent territories, or inaccessible arteries due to tortuous or small-caliber vessels [5, 6]. If not completely obliterated, dAVF can recur by drawing alternative feeder arteries via angiogenesis and dAVF-inducing factors [5,6]. The goal of TVE is complete occlusion of the draining sinus segment that is involved in the dAVF [4-6]. This will result in the complete obliteration of all fistulous points. Unfortunately, TVE is not always feasible, as the involved sinus segment can be challenging to access due to stenosis or venous sinus occlusion that occurs due to rapid arterial flow into the sinus from the fistula [6]. In these cases, alternative access to the fistula can be considered, such as a craniotomy for direct transcranial endovascular access.

## Case presentation

**Figure 1:**
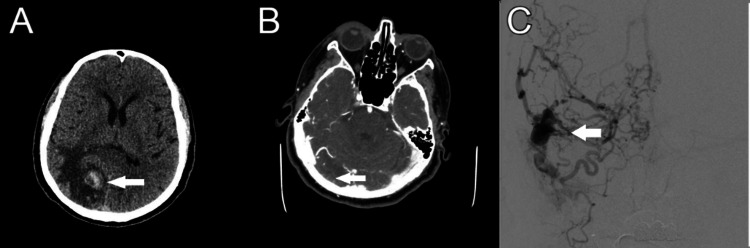
Selected initial neuroimaging A) Computed tomography head demonstrating right occipital hemorrhage with vasogenic edema. B) Computed tomography and angiography demonstrate a filling defect in the medial right transverse sinus and prominent cortical veins, which could be suggestive of underlying DAVF. C) Digital subtraction angiography demonstrating right transverse dAVF with cortical venous drainage.

A patient in their 60s presented to our facility with diplopia left homonymous hemianopia and severe headaches. The computed tomography head (CTH) showed a right occipito-temporal hemorrhage with surrounding vasogenic edema (Figure 1). Computed tomography angiography (CTA) identified dilated right hemispheric cortical veins with occlusion of the medial right transverse sinus and the right jugular bulb. A diagnostic cerebral angiogram identified bilateral indirect carotid-cavernous (CC) fistulae with retrograde drainage via the left superior ophthalmic vein (SOV) and a Cognard type IIa+b right sigmoid dAVF with multiple arterial feeders coming from branches of the right external carotid artery. Due to the occlusion of the medial right transverse sinus and right jugular bulb, there was extensive CVD. The superior sagittal sinus (SSS) drains via the left transverse and sigmoid sinuses.

Percutaneous transvenous endovascular access was unsuccessful via the right internal jugular vein (IJV) due to occlusion at the jugular bulb and via the contralateral left IJV and left transverse sinus due to occlusion at the right medial transverse sinus. The patient underwent TAE of the major feeding arteries with penetration of the right sigmoid sinus. Due to the tortuous and small-caliber feeding arteries, it was difficult to penetrate all fistulous connections. TAE resulted in diminished shunting without fistulous occlusion and a reduction in ecstatically draining veins. The patient's headaches improved, but visual symptoms continued.

Five months later, he developed right-sided pulsatile tinnitus, left ocular proptosis with chemosis, and left cranial nerve (CN) 6 palsy. Repeat angiograms demonstrated recruitment of the right sigmoid dAVF from the right occipital artery and increased shunting at the left CC fistula through the right SOV. Since he failed TAE and the right sigmoid sinus was isolated and inaccessible through the percutaneous route for TVE, we decided to proceed with craniectomy for transcranial endovascular embolization. 

**Figure 2:**
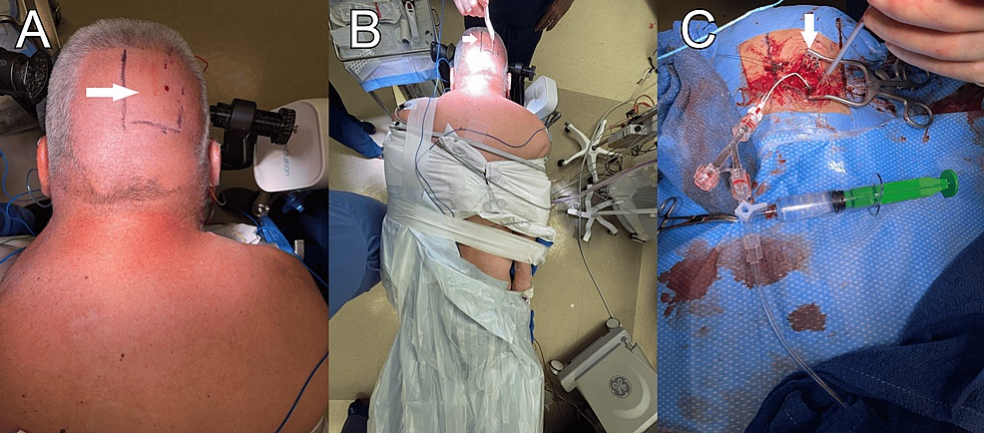
Craniotomy planning and incision A) Incision planning of the right transverse. B) Positioning with the right wrist exposed for angiography. C) Intraoperative set-up with endovascular sheath in the right transverse sinus.

The patient was taken to the OR and placed in a prone position with palms facing up (Figure 2). The Stealth neuronavigation system was used to locate the right transverse sinus relative to the sigmoid sinus and torcula. A transverse incision was made over the right transverse sinus. A 3-4 cm craniectomy was performed to expose the right transverse sinus. Two mobile C-arm fluoroscopy units were brought in for angiography. The right wrist was prepped, and the right radial artery was accessed with an introducer sheath. A glide catheter was navigated over a glide wire into the right common carotid artery for angiography. The transverse sinus was surgically opened, and we dissected laterally until some of the obstruction was freed. We then penetrated through it with a Rhoton 6 dissector and subsequently advanced a short 4-French sheath. The sheath was tunneled into the sigmoid sinus under fluoroscopic roadmap guidance, and a muscle flap was sutured to control bleeding around the sheath. The sheath was then sutured to the skin edge and connected to a rotating hemostat valve.

**Figure 3:**
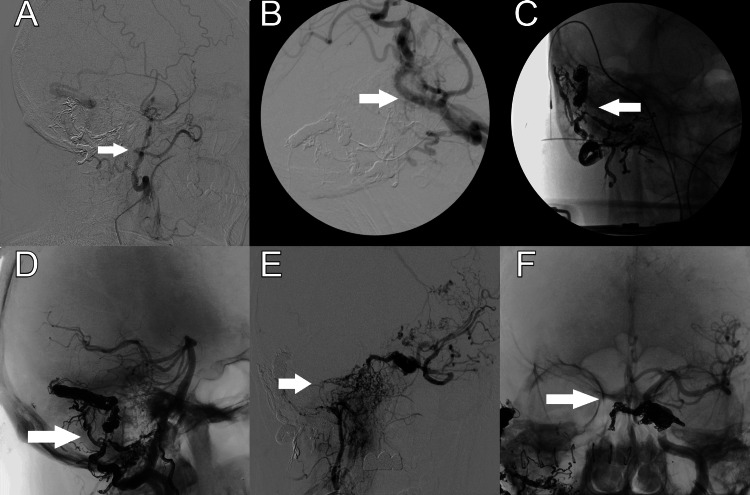
Selected operative digital subtraction angiograms A pre-operative angiogram with persistence of the right dural arteriovenous fistula (dAVF) after trans-arterial embolization. B) intra-operative embolization of dAVF using C-arm fluoroscopy. C) Post-embolization image using C-arm fluoroscopy. D) post-embolization angiography demonstrating dAVF occlusion. E) Carotid-Cavernous (CC) fistula pre-embolization. F) CC fistula post-embolization.

Under roadmap guidance, a microcatheter was navigated caudally into the sigmoid sinus. The sinus was embolized with platinum coils and Onyx 34 until there was a complete occlusion of the fistula (Figure 3). The dura along the superior aspect of the transverse sinus overlying the tentorium was then skeletonized. The open transverse sinus was packed with Surgicel, and a small piece of muscle harvest was sutured over to minimize the risk of venous bleeding. Cranioplasty was performed, and the wound was closed.

The patient was then taken to the angiography suite for treatment of the CC fistula. Drainage of the left CC fistula had remodeled due to a new occlusion of the left SOV and was rerouted via superficial cortical and deep veins. We performed percutaneous TVE of the bilateral CC fistulae, resulting in complete occlusion. After achieving complete occlusion of the multiple fistulas, a new left sigmoid sinus Cognard type I dAVF was visualized. 

The patient’s postoperative course was uncomplicated. He was discharged home in stable condition three days post-operatively. He had a complete resolution of symptoms at follow-up two weeks after discharge. At 12 months postoperatively, he was doing well clinically without any recurrence or new symptoms. He was offered a repeat follow-up angiogram but declined.

## Discussion

Transcranial endovascular embolization for dAVF is an uncommon procedure that has been described infrequently in the literature. Due to high venous output-induced veno-occlusive venopathy, percutaneous TVE can be difficult. TAE is an option in such cases; however, it can be difficult to achieve complete obliteration in cases with multiple complex arterial feeders. Failure to achieve complete obliteration can result in re-vascularization of the fistula, with continued risk for serious neurological consequences [4-6]. The use of flow-diverting stents has been debunked in the treatment of DAVF and results in recanalization and recruitment.

Our institution does not have a hybrid operating room (OR) angiosuite, so we performed the transcranial TVE with two mobile C-arms in the OR. This was beneficial because our neurosurgeon had access to his tools if he needed to surgically intervene. The downsides to using a mobile C-arm in the OR are poor image quality and slow frame rates. We found this set-up acceptable since the mobile C-arm could perform digital subtraction angiography and create a road map.

We decided to treat the right sigmoid sinus dAVF before the bilateral CC fistula because some of the drainage of the right sigmoid sinus dAVF was via medullary veins, which ultimately drained into the right cavernous sinus. Occluding the CC fistula first would have carried an elevated risk of increasing right hemispheric venous pressure and re-rupturing the right sigmoid dAVF.

It is crucial to re-evaluate the vascular drainage of the brain in the setting of multiple cranial fistulae after treating a single fistula. In our patient, the left SOV and left inferior petrosal sinus (IPS) had occluded, which resulted in retrograde cortical venous drainage and thus grade-up conversion of the bilateral CC fistula. It is unclear what resulted in the occlusion of the left SOV; however, consideration was given to the prone positioning during transcranial embolization. Regardless, we recommend that repeat angiography be performed after treating multiple complex dAVFs to confirm cure and reevaluate flow dynamics.

In our review of the literature, we found 23 publications detailing 61 cases of combined transcranial endovascular embolization of dAVF (Table 1) [7-29]. Forty-five of the 61 cases had an angiographic cure; however, not all cases had long-term follow-up. Twelve of the 61 cases reported complications, which ranged from recurrence of symptoms to death. The technical approaches varied significantly between the authors. 

**Table 1:** Details from prior published case reports M: male, F: female, ICH: intracranial hemorrhage, dAVF: dural arteriovenous fistula, CS: cavernous sinus, SS: straight sinus, TS: transverse sinus, TV-SS: transverse-sigmoid sinus, SSS: superior sagittal sinus, MMA: middle meningeal artery, DSA: digital subtraction angiography, TAE: transartial embolization, TVE: transvenous embolization, MRI: magnetic resonance imaging.

Year published	Reference	Patient age (years) and sex	Clinical presentation	dAVF location	Reason transfemoral unsuccessful	Surgical approach	Angiographic result	Clinical outcome
1989	Barnwell et al., Journal of Neurosurgery [7]	M mean age 56, F mean age 44	loud bruits, neurological deficits or intracranial hemorrhage	TS, SS, falx tentorial region, vein of Galen	inaccessible	Pre-operative TAE + surgery in 15 patients. Surgery included: 1. exposure of intracranial draining veins for TVE (9), 2. Exposure of intracranial feeding arteries to allow TAE (2) or 3. surgical resection (4)	For combined TVE: post op angiographic cure in 7/9 patients. TAE 1 with return of fistula.	TVE group 1 patient with hydrocephalus, 1 patient with repeat procedure, 1 patient developed venous sinus thrombosis and coma, 1 patient return of bruit but reported all doing well clinically. Combined TAE group with both with return of bruit. Surgical resection group with 1 occipital infarct
1991	Barnwell et al., Journal of Neurosurgery [23]	M 74, F 48	SAH, intracranial hemorrhage	SS, SSS	inaccessible	1. Suboccipital craniotomy with intra-op cannulation of posterior fossa vein with TVE 2. Craniotomy exposed 2 branches of middle meningeal artery which were cannulated and performed TAE	Both cases with obliteration of the fistula	3 year f/u reported good outcome
1996	Kasai et al., Neuroradiology [8]	80 M	seizure	TS	inaccessible	Initial TAE. Burr hole with cannulation of left transverse sinus for TVE	no recurrence angio at 1 year and MRI at 2 years	seizures well controlled, bruit no longer audible
1997	Pierot et al., Neurosurgery [11]	68 M	6 months hx of neurologic decline	SSS	Failed initial TAE.TVE inaccessible. Failed open surgery	Burr hole frontal region in front the SSS which was cannulated in OR and patient transferred to angio suite under anesthesia for TVE	Immediate and 1 month follow-up angiogram showed complete occlusion	Incomplete improvement of patient’s clinical status was seen in weeks after embolization. Patient died suddenly a few months which was assumed to be due to pulmonary embolism
1998	Kuwayama et al., American Journal of Neuroradiology [12]	48 M	tinnitus, headache, left hemiparesis, right frontal ICH	CS	inaccessible	Craniotomy performed and micro catheter introduced into sylvian vein for TVE	Immediately complete obliteration of the fistula. 2 day post procedure angiogram also showing complete cure of fistula	Tinnitus resolved post procedure
1999	Goto et al., Journal of neurosurgery [18]	12 patients: M and F, ages 36-66	seizure, cerebral edema, cerebral infarct, aphasia, SAH	TS-SS, CS	if TVE unable to be performed via transfemoral route	craniotomy with direct cannulation of diseased sinus with intraoperative embolization	5/12 had cure. 7/12 went on to have surgical isolation or resection.	Good clinical outcome. 2/5 patients with no adjuvant surgery with persistence of some of their presenting symptoms.
1999	Benndorf et el., Interventional Neuroradiology [13]	57 F	exophthalmos, chemosis, left eye pain, decreased visual acuity	CS	TAE partial treatment. TVE inaccessible	Pterional craniectomy with clipping and puncture of superficial sylvian vein. Catheter advanced through sphenoparietal sinus into CS and coiled	Immediate angiogram with no more filling of dAVF. Follow-up angiogram 5 years later with complete occlusion of the fistula and no signs of recannulation	Symptom improvement and recovery over 2 weeks
2002	Houdart et al., Journal of Neurosurgery [19]	10 patients: M and F, ages 21-70	cognitive impairment, headaches, IPH, seizure, infraction, tinnitus	SSS, TS	inaccessible	Craniectomy then 1-week later Sinus catheterization in the angiography suite.	all cases had angiographic cure	1 patient developed perioperative subdural hematoma. Clinically good result in all patients at 3 months: 5 patients with normal neurologic exams, 5 patients with improvement of clinical symptoms.
2004	Zink et al., Journal of Neuro Imaging [10]	65 F	Cerebellar hemorrhage with obstructive hydrocephalus	SS	inaccessible	Multistage 1. Percutaneous TAE some feeders Stage 2. 4 days later TAE anterior circulation feeders Stage 3. Hospital day 11 occipital craniotomy with straight sinus cannulation for TVE. Access tract was closed with Floseal.	Post embo angio with complete obliteration of the fistula. 6 month follow-up angiogram with persistent occlusion of fistula	Developed immediate hydrocephalus requiring EVD. 6 month follow up her neurologic symptoms had improved and she was living independently able to perform all activities of daily living
2006	Kong et al., Surgical Neurology [26]	5 patients, M+F, 45 to 69	exophthalmos with chemosis, headache, hemorrhage, venous infarct, seizure, aphasia	SSS, TV-SS	failed TAE, TVE inaccessible	Large burr hole or craniotomy overlying sinus with sinus catheterization for TVE in 4 patients, cranitomy with intracranial arterial catheterization with TAE	complete recovery on all	follow-up 6.5 months with significant improvement in all patients and no complications related to treatment
2008	Bruneau et al., Surgical Neurology [9]	58 F	acute coma from right cerebellar ICH with hydrocephalus	TV-SS	inaccessible	Image guidance located left transverse sinus. Craniectomy exposed sinus and the distal portion of the DAVF. Then the patient was moved to the angiography suite under the same anesthesia and placed in supine position. Using fluoroscopic guidance and road mapping the transverse sinus was punctured for TVE.	Immediate angiography with complete occlusion. 2 Month angiogram confied complete occlusion	Reported uneventful post-operative course
2011	Hurley et al., Neurosurgery [16]	75 F	Left temporal ICH.	CS	TAE aborted due to embolization material migration. TVE inaccessible	left orbitozygomatic craniotomy and left sylvian fissure vein was followed down to its attachment to the dura of the greater sphenoid wing catheterized for TVE	Immediate angio with occlusion of fistula and 1 week follow-up angiogram	Post-operative course uneventful. 6 Month follow-up she was neurologically intact and at cognitive baseline
2012	Chaudhary et al., Operative Neurosurgery [14]	82 F	visual changes, periorbital swelling	CS	TAE partial treatment. TVE inaccessible	Frontotemporal craniotomy performed, intraoperative left ECA roadmap used to identify superficial middle cerebral vein in the left sylvian fissure and temporal branch was isolated and catheterized for TVE	Immediate angiogram and venogram showed no flow through fistula. 18 month angio follow-up with partial recurrence	18 month follow-up chemosis resolved, estropia improved and visual acuity was stable. Persistent mild elevation of intraocular pressures
2012	Liu et al., Acta Neurochirurgica [28]	74 F, 75 F, 61 M	worsening aphasia, right hemiparesis and seizures, headache, ataxia, mental status changes	TV-SS, SSS	inaccessible	Left retromastoid craniectomy exposed left transverse sigmoid sinus and was catheterized. Angiogram performed using mobile C arm fluoroscopy in the OR and TVE	Immediate post-embolization with complete occlusion.	1 patient required long term AED to control seizures. No further clinical events after follow-up Mean 37 months (range 20-58 months)
2014	Kumar et al., Neurology India [27]	65 M	Headache, speech difficulties	TS	inaccessible	retrosigmoid craniectomy using neuronavigation and indocyanine green angiography, direct access of left transverse sinus	Immediate angio with cortical reflux reduced to 1 small vein. No follow-up angiogram	Improvement visual symptoms POD#1. No neurologic deficits 3 weeks post operatively
2014	Caplan et al., BMJ Case Reports [24]	55 F	Left sided pulsatile tinnitus, concentration difficulties, short term memory difficulties	TS	inaccessible	Brain lab system used for neuronavigation and intraoperative angiography used to localize the transverse sinus for incision and craniotomy. Single burr hole made over transverse sinus and left transverse sinus was catheterized for TVE.	Immediate angio with completed occlusion of fistula	2 week follow-up had improvement in symptoms and complete resolution of pulsatile tinnitus. At 3 month follow-up she returned to her baseline
2015	Shen et al., Journal of NeuriInterventional Surgery [20]	72 F	Left cerebellar ICH	Periclival venous plexus	inaccessible	Single stage procedure in Hybrid OR angio suite room. Pterional craniotomy performed and sylvian fissure split and posterior clinoid process approached. Doppler and Intraoperative angiography identified periclival venous plexus that was catheterized for TVE	Intraop angio with successful embolization	Reported recovered well with no complications
2015	Cowie et al., British Journal of Neurosurgery [21]	68 F 82 F	progressive cognitive decline, headache, vomiting, visual change	TS	TAE partial treatment. TVE inaccessible	Patient 1 had 2 stage procedure: Burr hole placed overlying sinus with cannulation. Endovascular sheath was held in place with a fenestrated titanium burr hole cover and suture, was taken to angio suite and had TVE then sheath removed. Patient 2 had burr hole in angio suite and Camino device inserted which the TVE was performed through	cure	reported good outcome for both patients at 3 month follow-up
2017	Park et al., Journal of Cerebrovascular and Endovascular Neurosurgery [29]	71 F	ICH, global aphasia	TS	inaccessible	Retromastoid craniotomy around the TS-SS junction. TCD was used to identify the location of the sinus that was catheterized. Under fluoroscopy in the OR TVE was performed	Immediate angiogram with complete occlusion. 1 week post op angio showed no recannulation	reportedly uneventful and some improvement in aphasia
2017	Akamatsu et al., World Neurosurgery [17]	84 F	ICH	CS	planned as single procedure with emergent hematoma evacuation	Frontotemporal craniotomy performed, SMCV identified using right external carotid angiography and doppler-flometry, arterialized SMCV isolated and catheterized. C-arm fluoroscopy and right ECAG was to guide the micro catheter that was advanced into the cavernous sinus for TVE	Immediate angiogram with complete fistula occlusion. Angio 5 weeks post op no recurrence	No progressive symptoms, no rebleeding post-operatively. Just persistent left hemiparesis from the ICH
2019	White et al., Operative Neurosurgery [22]	66 F	subacute confusion	TS	risky access through traditional route	Hybrid OR-Angio suite. Angio left external carotid used to visualize and used to guide access. Drilled used to decorticate the outer cortex to expose diploic vein that was catheterized for TVE	Immediate angiogram with occlusion. 3 Month angiogram confirmed persistent complete occlusion and no residual	Symptoms improved POD 1. 20 months post embolization follow-up was symptom free
2019	Fioravanti et al., Journal of Neurosurgical Sciences [15]	39 M	proptosis and chemosis of right eye	CS	inaccessible	Right pterional approach to exposure of arterial sylvian vein was punctured and the CV was catheterized for TVE under fluoroscopic guidance in the OR. After coiling surgical cauterization of the superficial sylvian vein, temporo-polar vein and medial sphenoparietal sinus was performed	occluded 3 days post op MRA and 10 days DSA confirmed occlusion	Resolution of exophthalmos and chemosis without neurologic sequelae and reported excellent clinical outcome at 6 month.
2022	Hoffman et al., World Neurosurgery [25]	unspecified age F	Nausea, vomiting, dizziness	Tentorial dAVF	TAE attempted and failed	Temporal craniectomy over MMA and 4F sheath placed. Selective angiography of MMA and micro catheter navigated to fistula and embolized with onyx. Video of technique provided online	Immediate complete occlusion.	immediate post-operative at neurologic baseline

Combined neurosurgery and endovascular approaches to intracranial vascular malformations became known after Mickle et al. published “transtorcular embolization of the Vein of Galen Aneurysm” in 1986 [30]. Since then, their technique has been modified and used for the combined transcranial endovascular treatment of complex intracranial dAVF. 

Barnwell et al. published their experience with the management of complex intracranial dAVF [7]. They described 15 patients with dAVF who failed to respond or had lesions that were inaccessible to conventional approaches. The combination therapy they performed included craniotomies with transcranial TVE (nine patients), craniotomies with transcranial TAE (two patients), and open surgical resection (four patients). In the transcranial TVE subgroup, 7 of 9 patients achieved post-op angiographic cure. Clinically, four patients in this subgroup suffered complications. One patient developed transient coma related to embolic material flowing into the ponto-mesencephalic vein and post-operative venous sinus thrombosis. This patient recovered from anticoagulation use. Another patient in this group developed communicating hydrocephalus that required shunting. One of the patients required a repeat procedure for a cure. Their study included two patients in the series who underwent craniotomies for transcranial TAE. One of the patients in the TAE subgroup had a fistulous recurrence. The authors concluded that surgical exposure to allow for intracranial endovascular access in select cases is safe and effective. 

Kasai et al. published a report of an 80-year-old male who presented with seizures and was found to have a dAVF [8]. There was a thrombus on the distal and proximal ends of the draining fistula, which prevented TVE. TAE was attempted but did not result in a cure. They performed transcranial endovascular embolization. Good clinical and angiographic outcomes were reported after two years of post-treatment follow-up. Cases with similar circumstances were reported by Zink et al. and Bruneau et al. [9,10]. In the report by Zink et al., the patient developed hydrocephalus, requiring an extra-ventricular drain immediately after removing the vascular sheath used for embolization. However, the patient reportedly had a good clinical outcome at six months post-treatment. 

Pierot et al. reported a case of dAVF of the superior sagittal sinus (SSS) with venous sinus thrombosis of the posterior aspect of the SSS who presented with a coma following a lumbar puncture [11]. They attempted TAE with transient symptom improvement, but the fistula remained and developed extensive arterial collateralization. Surgical sinus skeletonization was attempted without success. They proceeded with a combined surgical and endovascular treatment. The patient was taken to the operating room (OR) for exposure to the SSS with a burr hole and cannulization. The patient was then transferred, while under general anesthesia, to the angiography suite for embolization. A post-operative angiogram demonstrated complete occlusion one month later. The patient had an incomplete recovery of his symptoms and suddenly died months after the operation from a suspected pulmonary embolism. 

Kuwayama et al. reported a case of CC fistula that couldn’t be treated via standard measures [12]. The patient underwent craniotomy with cannulation of an arterialized sylvian vein for endovascular embolization. The patient had symptom improvement and an angiographic cure by postoperative day two. Benndorf et al., Hurley et al., Chaudhary et al., and Fioravanti et al. reported similar cases with good long-term outcomes [13-16]. Akamatsu et al. presented a similar case in 2017 that was performed at the junction of an emergency hematoma evacuation in the OR using a mobile C-arm fluoroscopy [17]. They reported an angiographic cure of the fistula at five weeks post-embolization angiogram.

Goto et al. published their reports of combined endovascular and surgical treatment of dAVF in 17 patients [18]. They provided a detailed treatment protocol that they followed. They would first perform TAE, followed by TVE, within a few days. If transfemoral TVE was not possible, then they performed transcranial TVE. If the fistula persisted after TAE and TVE with venous reflux, then they proceeded with surgical isolation or resection. Twelve of the 17 patients included in the series underwent intraoperative embolization. Five of the 12 patients had angiographic cures. However, seven of the 12 patients went on to need surgical treatment. Of the five patients who had an angiographic cure with intraoperative embolization, two had persistence of their presenting neurologic deficit at follow-up six and eight years post-treatment. 

Houdart et al. published their case series of 10 patients who underwent transcranial endovascular embolization, including their technical recommendations [19]. In their first case, they attempted to catheterize the sinus in the OR immediately after craniectomy; however, this was complicated by a subdural hematoma (SDH). Thus, the other nine patients performed venous sinus catheterization one week post-craniectomy in the angiography suite. They had to take seven of their patients back to the OR for enlargement of the craniectomy site. The authors suggested that the craniectomy should expose 5-8 cm of the underlying sinus to make the catheterization straightforward. At three-month follow-ups, five patients had normal neurological examinations, and five patients had symptom improvement.

Shen et al. published their case of a patient with a cerebellar dAVF that was drained by the periclival plexus [20]. They performed the procedure in a hybrid OR that was equipped with a robotic angiographic fluoroscopy system. They performed a pterional craniotomy with dissection of the sylvian fissure and direct catheterization of a small vein draining into the periclival venous plexus, which was used for TVE. They reported an angiographic cure for the fistula, and the patient had a good clinical recovery. 

Cowie et al. published their experience with two cases [21]. In one case, they performed a two-stage procedure with a burr hole made with a beveled anterior edge, followed by a micropuncture technique to insert a four-French endovascular sheath. A fenestrated titanium plate was screwed into place with the endovascular sheath exiting through the fenestration, and the scalp was sutured closed. Afterward, the patient was transferred to the angiogram suite for transcranial embolization, and the endovascular sheath was removed after completion of the procedure in the angio suite. In their other case, they performed a single-stage procedure completely in the angiography suite. Fluoroscopy was used to identify the draining right transverse sinus, and a burr hole was made so that a Camino small-bore cranial access device could be placed. A peripheral venous catheter was inserted through the lumen of the Camino device, and a microcatheter was advanced for transcranial embolization. Both patients reportedly did well without complications.

White et al. reported a successful case of transcranial endovascular embolization of a dAVF along the sphenoid ridge using a novel approach. Instead of a full-thickness craniotomy, they decorticated the outer cortex overlying a calvarial diploic vein, which was then cannulated for TVE. This technique resulted in an angiographic cure, with the patient remaining clinically well at 20 months post-embolization [22].

## Conclusions

Our literature review shows that the transcranial embolization procedure has been infrequently performed over the past 30 years. Based on our experience and review, transcranial endovascular embolization appears to be feasible and likely safe. The true risks of the procedure are unknown since all published reports in the literature were just single cases or small case series. More research is needed to determine the optimal and safest way to perform combined transcranial endovascular embolization. More efforts in equipment design should be made to develop technologies specifically for transcranial endovascular embolization. Advances in research and technology in combined open surgical and endovascular techniques could extend the reach of endovascular neurosurgery and give options to patients with “untreatable” lesions. Transcranial TVE of dural arteriovenous fistulas is occasionally necessary for the treatment of complex dAVF. This appears to be a reasonable and feasible approach to the management of high-grade dAVF that cannot be adequately treated through the traditional percutaneous route.
